# Limb phase flexibility in walking: a test case in the squirrel monkey (*Saimiri sciureus*)

**DOI:** 10.1186/s12983-019-0299-8

**Published:** 2019-02-18

**Authors:** Charlotte Elizabeth Miller, Laura Elizabeth Johnson, Henry Pinkard, Pierre Lemelin, Daniel Schmitt

**Affiliations:** 1 Centre for Applied Anatomy, University of Bristol, Southwell Street, Bristol, BS2 8EJ UK; 2 Department of Pathology and Anatomical Sciences, University of Missouri, Missouri, USA; 3 Center for Computational Biology, University of California, Berkeley, California USA; 4 Division of Anatomy, Department of Surgery, Faculty of Medicine and Dentistry, University of Alberta, Edmonton, Alberta Canada; 5 Department of Evolutionary Anthropology, Duke University, Durham, North Carolina USA

**Keywords:** Gait, Duty factor, Speed, Locomotion, Biomechanics, Arboreality, Primates

## Abstract

**Background:**

Previous analyses of factors influencing footfall timings and gait selection in quadrupeds have focused on the implications for energetic cost or gait mechanics separately. Here we present a model for symmetrical walking gaits in quadrupedal mammals that combines both factors, and aims to predict the substrate contexts in which animals will select certain ranges of footfall timings that (1) minimize energetic cost, (2) minimize rolling and pitching moments, or (3) balance the two. We hypothesize that energy recovery will be a priority on all surfaces, and will be the dominant factor determining footfall timings on flat, ground-like surfaces. The ability to resist pitch and roll, however, will play a larger role in determining footfall choice on narrower and more complex branch-like substrates. As a preliminary test of the expectations of the model, we collected sample data on footfall timings in a primate with relatively high flexibility in footfall timings – the squirrel monkey (*Saimiri sciureus*) – walking on a flat surface, straight pole, and a pole with laterally-projecting branches to simulate simplified ground and branch substrates. We compare limb phase values on these supports to the expectations of the model.

**Results:**

As predicted, walking steps on the flat surface tended towards limb phase values that promote energy exchange. Both pole substrates induced limb phase values predicted to favor reduced pitching and rolling moments.

**Conclusions:**

These data provide novel insight into the ways in which animals may choose to adjust their behavior in response to movement on flat versus complex substrates and the competing selective factors that influence footfall timing in mammals. These data further suggest a pathway for future investigations using this perspective.

## Background

The selective factors influencing footfall timings in quadrupedal animals are a topic of long-standing debate, with most of the discussion focusing on the difference between primates and other mammals, and the potential mechanical consequences of footfall timings for either energy expenditure (defined broadly as patterns that affect muscular effort) or stability (defined broadly as a way to avoid falling [1–12]). Previous research has made significant strides and laid a valuable foundation for understanding the effects of footfall timing on mammalian gait mechanics, especially the differences between diagonal- and lateral-sequence patterns (DS and LS, in which contact of the right hind limb is followed by the contralateral or ipsilateral forelimb respectively [13]) and their specific implications for primate evolution.

Despite the extensive work in this area, several issues remain less well explored. Specifically, the effect of footfall timings on mechanical stability and mechanical energy exchange on arboreal and terrestrial substrates have rarely been considered together in a single model. As a result, the ways in which these potentially competing demands interact in determining footfall timings in mammals is still poorly understood. Here we combine some of these factors in a model that attempts to predict how tendencies toward certain footfall timings are achieved during walking strides. An additional goal is to consider how substrate type–branch vs. ground–interacts with these factors. We then use a small empirical dataset of footfall timings from a primate capable of using different footfall patterns to provide an initial test of the utility of the model, and establish a pathway for further study using this perspective.

In order to develop the logic for the model and its value in addressing questions about locomotor behavior, we include in the following subsections details about variables included in the model, predictions for those variables, and also a review of studies relevant to understanding limb phase values on different substrates. The hypotheses follow those subsections.

### The basis of the model for limb phase

During walking strides, limb phase values (the percentage of the stride cycle between the touchdown of a hindlimb and its ipsilateral forelimb) of 25% or 75% (what are often called ‘singlefoot’ gaits [13]) should provide the most consistently stable base of support, maximizing the proportion of the stride supported by three limbs [3, 5, 13, 14]. However, empirical data generally cluster around limb phase values of 15 and 65% [5, 14–19], suggesting that some other mechanism is involved in moving animals away from these stable singlefoot gaits. One of the factors that may influence this choice is mechanical energetic cost.

Direct measures of mechanical energetic cost are not easy to collect for individual locomotor behaviors, especially in the field; however, several mechanical features linked to an animal’s potential for energetic expenditure have also been linked to footfall timing in quadrupeds. Energetic recovery (i.e., the percentage of kinetic energy (KE) reclaimed from the phase relationship between KE and gravitational potential energy (PE)) of the center of mass (COM) during a stride, can reduce muscular effort to accelerate and decelerate the COM. This has been noted to peak at around 20% limb phase in walking cats [18], consistent with (and central to) one hypothesis related to modelling a walking quadruped as the interaction between two independent sets of inverted pendulums [14]. By fixing the distance between the interacting pendulums in a four-bar-linkage model (much like adding an inextensible back segment), work by Usherwood et al. [17] has gone on to estimate that at a similar value of footfall timing, redirections of the COM (collisions [20, 21]) are also minimized.

**Fig. 1:**
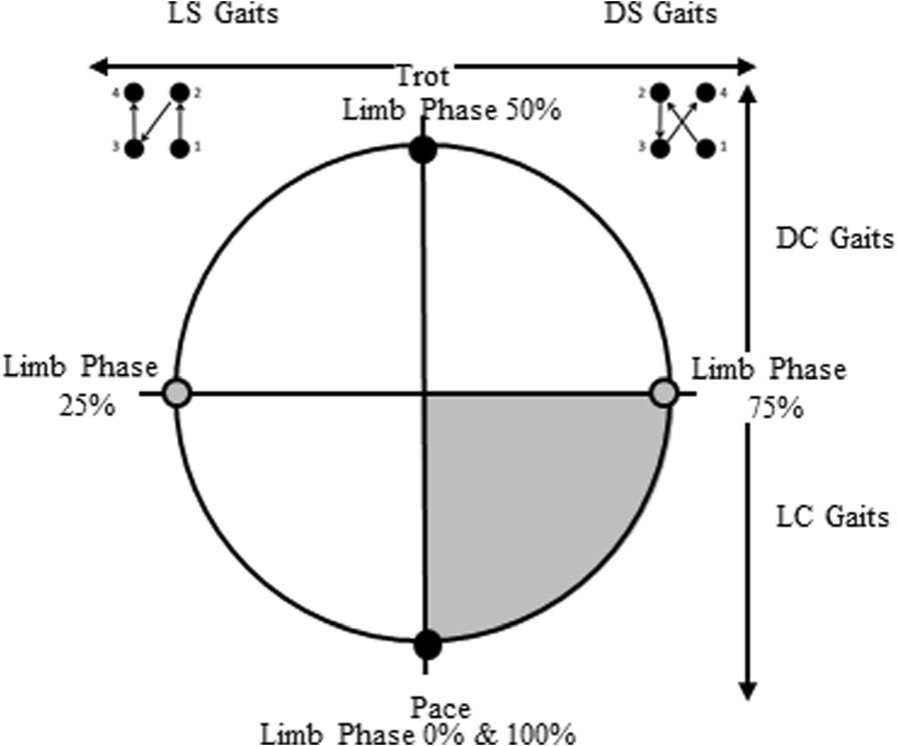
The phase wheel. Limb phase values from 0 to 100% (where 0% = 100%) can be represented as a circular continuum (a circular representation of the y-axis of a classic Hildebrand diagram). The circle can be segmented into four quadrants, each running between a simultaneous footfall pattern—a pace or a trot (black circle), and an evenly spaced footfall pattern—what Hildebrand [13] called the singlefoot (gray circle). The circle can be divided into halves vertically and horizontally by sequence and couplets respectively in which LS: lateral sequence, DS: diagonal sequence, LC: lateral couplet, DC: diagonal couplet; the shaded gray area is that of the DSLC gaits which appear to be uncommon in nature, see text for details

Alternatively to or in connection with energetic concerns, researchers have argued that certain footfall timings enhance stability. In one of the most recent formulations, the support polygon model of Cartmill et al. [5] hypothesizes that an animal moving on narrow and unstable supports will tend to reduce the proportion of the stride during which it is supported only by two limbs on the same side of the body. Such unilateral bipedal support is greatest in the pace (0 or 100% limb phase), and least in the trot (50% limb phase), leading Cartmill et al. [5] to argue that certain footfall patterns provide greater stability than others on arboreal supports (DS versus LS, see below; Fig. 1).

These two types of studies–those concerned with energetic costs, and those concerned with stability–form the foundation of the model presented here. If this model is robust, it will allow researchers to infer underlying mechanical factors from data on footfall timing that can be more easily collected in the field (see D’Août and Vereecke [22] for such approaches) and allow for broader studies of mammalian gaits.

### The variables included in the model

#### Limb phase

Footfall sequence can be defined by values of limb phase: the percentage of the stride cycle between the touchdown of a hindlimb and its ipsilateral forelimb ([13] Fig. 1). Limb phase values range from 0 to 100%, where both 0 and 100% represent the lateral-sequence pace in which ipsilateral (same side) pairs of fore- and hindlimbs strike the substrate simultaneously. A limb phase value of 50% represents the trot, in which contralateral pairs of fore- and hindlimbs strike the substrate simultaneously. Hence, the pace and trot can be classified as “simultaneous gaits”.

Limb phase values of 25 and 75% indicate that all four footfalls are equally spaced in time (the “singlefoot” of Hildebrand [13]). Singlefoot gaits with limb phase values of 25% follow what is called a lateral-sequence (LS) footfall pattern, in which right hind (RH) contact is followed by right fore (RF), then left hind (LH), and left fore (LF). Singlefoot gaits with limb phase values of 75% follow the diagonal-sequence (DS) footfall pattern with the following sequence: RH, LF, LH, RF. Any gait with non-simultaneous hind to fore footfall timings can be classified as LS or DS. LS gaits occur between 0 and 50% limb phase, and DS between 50 and 100%, although limb phase values above 75% have only very rarely been observed in nature [5].

Limb phase as used here is identical to the value plotted on the y-axis of a Hildebrand diagram [4, 13], gait number [6, 9], or the term diagonality employed by Cartmill and colleagues [5, 7, 23, 24]. Here we use the term limb phase for clarity, as the ‘most diagonal’ gait is the trot, at a limb phase of 50%, hence above this value DS gaits become less, not more, diagonal (see Fig. 1).

Footfall sequences can be further broken down by couplet timings that describe which limbs are in motion at roughly the same time, a value dependent upon which limb pairs strike the ground separated by less than 25% of total stride time. Lateral couplet (LC) gaits, in which ipsilateral limb movements are closely coordinated in time, occur between limb phase values of 0–25% and 75–100%, while diagonal couplet (DC) gaits, in which contralateral limbs are temporally coordinated, occur between limb phase values of 25–75%.

**Fig. 2:**
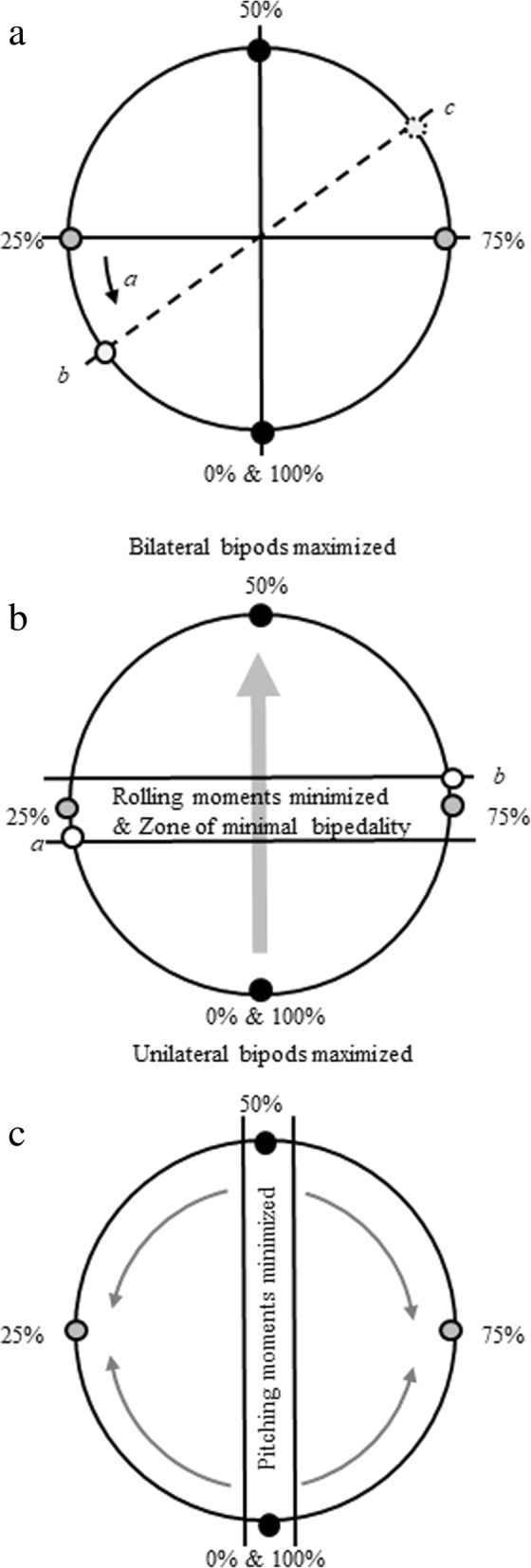
The limb phase constraint model. The wheel is split into quadrants as described in Fig. 1. Gray arrows represent increases, black arrows directions of constraints and tradeoffs in locomotor mechanics. **a**) Energetics. Arrow *a* represents the incentive to move away from the singlefoot gait to avoid limb interference, while point *b* indicates an approximation of the predicted value of greatest energetic exchange derived from the models and empirical data of Griffin et al. [14], and Usherwood et al. [17], based on pendular and collisional mechanics respectively (see text for details). Point *c* represents an extrapolation of this into the diagonal sequence, diagonal couplet (DSDC) footfall pattern. **b**) Roll. Following Cartmill et al. [5], limb phase values can be split into a ‘zone of minimal bipedality’ (where, in walking, tripods and quadrupods predominate, helping to reduce roll), and two zones dominated by unilateral bipods around the pace (0, 100%) and bilateral bipods around the trot (50%). Bilateral bipedality is thought to generate less roll than unilateral bipedality. The gray arrow represents the continuum of maximal to minimal proportions of unilateral bipods, and minimal to maximal proportions of bilateral bipods contained within strides at a given limb phase value. At the edge and outside of the ‘zone of minimal bipedality’ (open circles) a DSDC gait will contain a greater proportion of bilateral bipods (point *b***)** than the equivalent lateral sequence, lateral couplet (LSLC) gait (point *a*). **c**) Pitch. Pitching (both fore and aft) moments are minimized in a zone at the simultaneous gaits, increasing as limb phase values approach those of the singlefoot gaits

As seen in Fig. 2, a linear change in limb phase values from 0 to 100% (and hence footfall timings) involves several interacting oscillations in biomechanical effects, rather than progressive change. Shifting between simultaneous gaits (from pace to trot and back to pace, 0 to 50% to 100% limb phase values) through the sequenced gaits between those values will produce changes in both whole body mechanics (tendency to pitch and roll) and energetic costs (limb interactions affecting the muscular effort required to accelerate and decelerate the COM). To account for this and to express that change analytically and graphically, we treat limb phase as cycling around a ‘wheel’ of values (Fig. 1). It is separated into quadrants representing the four gait spaces. These quadrants are divided by the lines that represent the two simultaneous footfall gaits: the trot (50%) and the pace (0% or 100% limb phase values), and the two sequenced singlefoot gaits: the LS singlefoot (25% limb phase value) and the DS singlefoot (75% limb phase value), each of which is defined by the underlying mechanical properties of its footfall pattern and timings.

In the model presented here for walking gaits, the predictions concerning the tendencies of any given animal to adopt a particular limb phase result from the interacting demands associated with elements of potential for mechanical energy exchange and the need for on-branch stability. This is not to say that we believe these to be the only factors influencing footfall timing. Needs for stealth (reduced vertical movement of the COM to avoid notice) or low peak substrate reactions forces may also be important (see Schmitt et al. [16] for a discussion of both), but for simplicity our initial model assumes that these are both minimized by maximization of periods of tripedal support during the singlefoot gaits.

We also assume that reducing energy costs, where possible, is important on all substrates as a way to control energetic costs associated with locomotion. In our model, this factor is represented by the relationship between measures of percent recovery and collision reduction at limb phase values of 15–20 or 65–72% (Fig. 2a). Animals moving in a complex, arboreal environment, where tendencies to pitch and roll are higher because of the narrow support base (increasing the challenge posed by moments of the COM around the substrate [25]) and consequences of falling may be severe, animals may wish to maximize footfall patterns that minimize these deviations (Cartmill et al., [5, 7, 8] Fig. 2b).

Our initial model, therefore, recognizes that although certain limb phases may be more desirable from an energetic standpoint, mechanical constraints such as pitch and roll may lead animals to adopt gaits that are more energetically costly but more stable. This will vary, of course, depending on ecological context. Animals presented with a thin arboreal support, for example, may prioritize stability over energetic cost. That cost, which is not always the central priority of animal locomotion, is well understood (see [18, 26] for specific examples). However, this potential balance between stability and energy expenditure has not been included in previous models, and is rarely discussed in the context of arboreal and terrestrial substrate use.

#### Energetic cost

There is theoretical and empirical evidence that limb phase can influence the muscular effort needed to accelerate and decelerate the COM [14, 17, 18], and this cost may be an important selective factor in determining the limb phases used by an animal (Fig. 2a). The extent to which this cost is a central selective factor in animal locomotor behavior is an area of considerable debate. Whether animals consistently adopt postures and gaits that minimize energetic cost remains an empirical question and one that is driven by both ecology and body size. Some animals may prefer stealth or speed to a reduction in cost [18] or choose the shortest travel path rather than the cheapest gait [26]. It has also been noted that small animals may not be able to take advantage of energy recovery mechanisms like the inverted pendulum mechanics seen in larger animals [15, 27] and therefore select gaits that favour rapid accelerations and changes of direction. Nonetheless, both Griffin et al. [14] and Usherwood et al. [17] have presented theoretical and empirical data for dogs, suggesting that energetic cost may be a key factor in footfall timings. Thus, our model considers where on the limb phase continuum the cheapest footfall timings might be found (from the point of view of inverted pendulum mechanics, but with reference to possible requirements for reduction of collisional energy losses [17]). We propose to see if a model that incorporates factors of energetic cost can effectively predict tendencies for footfall timings in mammals other than dogs and on substrates other than flat ground.

With this perspective in mind, we hypothesize that, all else being equal, on all substrates and for all footfall sequences (LS and DS) animals should choose to use footfall timings that minimize energetic cost by reducing muscular effort. This is, of course, a simplification of the pressures experienced in nature. However, we do expect that energetic cost (in terms of external work on the COM) will explain the majority of variation in footfall timing in situations in which controlling other factors such as pitching and rolling moments is less important (such as walking on the ground).

In non-primate mammals, which tend to use LS gaits during walking, the least energetically costly limb phase values in terms of pendular exchange of kinetic and potential energy fall between the LS pace (limb phase > 0%) and LS singlefoot gait (limb phase < 25%), between values of about 15–22% (see Fig. 2a; [14, 17]). At these limb phase values, the COM of the entire animal oscillates like an inverted pendulum, effectively converting potential to kinetic energy, while simultaneously minimizing inelastic redirections of the COM (known as collisions), thus reducing the muscular effort required to control COM movements. The tendency for animals to select footfall timings in this ‘reduced cost zone’ between simultaneous and singlefoot gaits is limited by two factors (Fig. 2a, constraint a): (1) the need to reduce periods of locomotion during which fore- and hindquarter pendula may be working in opposition (reducing passive energy exchange at the COM [14]), and (2) the need to minimize collisional energy loss from redirections of the COM [17]. Because these two studies [14, 17] consider slightly different mechanical factors, the specific values for footfall timings that represent energetic optima differ slightly. Thus, for the purpose of this model, the ‘reduced cost zone’ is defined as limb phases that land between 15 and 22% (Fig. 2a).

When looking at primate footfall patterns we also need to consider the 50–75% phase values of the DSDC gaits, which were excluded from these earlier studies. Based on mechanical similarities with the 0–25% LSDC region, we theorise that there will be a corresponding ‘reduced cost zone’ above 50% in the region of the DSDC footfall pattern, whose lower bound is a simultaneous gait (the trot, limb phase 50%) and upper bound a singlefoot gait (limb phase 75%). We have extrapolated an equivalent ‘expected’ region of values at 65–72% for these DSDC gaits seen in primates (the 15–22% region of [14, 17] plus 50%) (Fig. 2a, point c, by extrapolation from point b).

It is important to note that the exact values at which maximal inverted pendular energy exchange occurs in primates, which use DSDC gaits regularly, may be affected by differences in limb length and force distribution between primates and other quadrupeds [14, 17]. A feature of the models and simulations in both Griffin et al. [14] and Usherwood et al. [17] is their use of a traditional ‘dog-like’ centre of mass, where more of the animal’s body mass is supported by the forelimbs than hindlimbs (approximately 60%, see [14, 17] for discussion of this value). Both studies recognise centre of mass location as a core value in their predictions of the limb phase at which ‘perfect’ pendular exchange may be attained, and that centre of mass position is central to moving this ‘perfect’ value away from the simultaneous gaits at 0 and 50% limb phase. They then go on to account for the variations seen in empirical data from dogs. Usherwood et al. [17] attributed this variation to avoidance of energetic loss from collisions, which will be greater in the simultaneous gaits where two limbs strike the ground at the same time, but will be reduced when footfalls are more separated in time [20, 28].

Primate mass distribution is a somewhat complex question. Calculations from cadaveric studies suggest that in animals ‘posed’ in a standing position, mass distribution is similar to these ‘dog-like’ values seen in non-primate mammals, however, measurements from ground reaction force data suggest a ‘dynamic’ center of mass skewed towards greater weight support by the hindlimbs [3]. For this reason we do not provide an explicit expected value for pure, ‘perfect’ pendular exchange in primates, but rather a ‘zone’ of values based on the more general conclusions derived from Griffin et al. [14] and Usherwood et al. [17]. Studies reporting comparable empirical data from primates moving on ‘unchallenging’ substrates give comparable phase values to those observed in dogs, and to those we predict from our theoretical extrapolation to the diagonal-sequence sectors. Schmitt [19] previously noted that primate data collected by Cartmill et al. [5] often clustered near limb phase values of 65%. In addition to that, studies of capuchin monkeys [11, 29] demonstrated that this primate tends towards using singlefoot footfall patterns, as predicted by our model, with LS and DS values distributed between 15 and 30% limb phase and 60–80% limb phase respectively. On a flat surface, ring-tailed lemurs have been observed attaining a maximum degree of pendular recovery values similar to those of walking dogs (71%) at a mean limb phase of 64% [30], which is comparable to our predicted diagonal-sequence values.

#### Mechanical stability

We expect that minimizing rolling and pitching moments, while important for all forms of locomotion, will be more important on arboreal substrates. In this context, it is predicted that animals will reduce their use of footfall patterns that favour energetic cost reduction and increase those that favour this specific type of mechanical stability. The extent to which animals will choose to adopt pitch- and roll-reducing limb phases should vary further depending on whether the support is a simple long branch with no laterally projecting side branches or complex with multiple side branches, the latter providing handholds that increase the base of support and reduce rolling. Thus, on the ground and complex branched substrates, the hands and feet can be placed with a broader gauge (away from the trunk), reducing both the magnitude and effect of COM moments around the substrate, and reducing the need for footfall sequences that reduce roll.

Rolling moments (torques around the sagittal body and substrate axes; see [31]) are an important factor on substrates that are narrow relative to an animal’s body width, which arboreal habitats very often are [25] as they can result in injurious or deadly falls. Cartmill et al. [5, 7] argued for a direct relationship between the magnitude of rolling moments and the proportion of any given stride when body weight is supported by two limbs on the same side of the body, described as unilateral bipods. Longer periods of unilateral bipedal support increase rolling moments, which increase the muscular effort required to resist them, and increase the likelihood of falling off the branch. In contrast, bilateral bipods (i.e., two supporting limbs on opposite sides of the body) are better able to resist rolling moments. Better still in terms of the ability to resist rolling moments is tripedality, which is more prevalent at slow speeds, especially during LSDC gaits, as per the Support Polygon Model of Cartmill et al. [5, 7].

The Support Polygon Model model sets boundaries for factors such as diagonal bipedality, within which gaits should cluster to maintain dynamically stable support patterns. The boundaries of the roll-related aspects of the Support Polygon Model (those focusing on periods of bipedal support) as calculated by Cartmill et al. [5] can be translated to our limb phase value model (Fig. 2b). Thus, around the singlefoot walking gaits, there is a ‘zone of minimal bipedality’ within which periods of tripedal support are maximized. The exact values spanned by the ‘zone of minimal bipedality’ (which depends upon duty factor and hence speed) will therefore be those at which rolling moments will also be minimized. Simultaneous gaits have the largest proportion of the stride supported by only two limbs, ranging on a continuum from maximized unilateral bipods (relatively high rolling moments) in pacing to maximized bilateral bipods (relatively low rolling moments) in trotting. Extended periods of support by only two limbs will increase rolling moments compared to gaits with periods of support by three limbs. Hence, both the trot and the pace will have greater rolling moments than those sequenced gaits in the ‘zone of minimal bipedality’ in which periods of tri- and quadrupedality are maximized. However, because unilateral bipods are still the least stable according to the Support Polygon Model, rolling moments will be greater in pacing than they are in trotting. For values falling outside (or at the edge) of the ‘zone of minimal bipedality’, DSDC gaits will, therefore, have a greater proportion of diagonal bipods than LSLC, and hence less roll [7].

Pitching moments (rocking back and forth) are a fundamental part of generating forward motion. However, pitching moments must be maintained at levels which safely prevent toppling forward or backward during locomotion. In arboreal animals, mitigating pitching moments is particularly important in forward walking along a branch from its origin on the trunk to its end, as it will most likely taper down and become more compliant. Without control of forward pitching moments, the animal will be more likely to fall forwards as the branch bends or even breaks [7].

Unlike rolling, pitching moments are minimized in simultaneous gaits for which hind- and forelimbs land simultaneously and can resist forward pitch. Pitching moments become more pronounced as limb phase values approach those of singlefoot gaits (Fig. 2c). It has been argued that DSDC gaits allow the COM to be in line or near the grasping hindfoot as the contralateral forefoot touches down on an untested support [5, 7, 23], while at the same time minimize periods of unilateral bipedality [7]. If the support does give way under the animal’s weight, DSDC gaits promote backwards pitch, enabling the animal to pull back by virtue of its grasping hindfoot (or prehensile tail; [5, 7, 23, 24]).

### Previous studies

Despite the potential importance of mechanical factors such as pitch and roll in the evolution of footfall patterns and arboreal locomotion, few studies have compared variation in limb phase for more than one substrate type in committed arboreal animals such as primates, although those that do provide critical insight into the mechanics of footfall patterns. The debate about why primates and other mammals choose DS versus LS gaits is well trodden and does not need to be re-debated here (for an overview see [3, 5–7, 10, 32–36]). Instead, here we focus on developing a generalized model for walking footfall patterns and timings as they exist on a *continuum*. However, a number of studies provide data or concepts that are relevant to our model, and potential methods for testing its predictions.

Many previous studies have focused on ‘ground-like’ supports such as a wooden runway or plank [6, 33, 34, 37, 38]. Others have focused on the support favored by the animal; ground for terrestrial animals, pole for arboreal ones [5, 10, 36], without collecting directly comparable data on both terrestrial and arboreal supports for all species. Differences between locomotion on a flat surface (ground) and a straight pole have been recorded in the same animals for the same set of experiments in capuchin monkeys [11], squirrel monkeys in both the lab and the wild [39], mouse lemurs [40], opossums [23], sugar gliders [41] and kinkajous [24], and between large and small pole or branch diameters for a sample of strepsirrhine primates [42], wild tamarins [12], the feathertail glider [43] and several rodent species [44–46].

Lateral branch projections from the main branch of a tree (referred to here as side branches) are known to be used as hand and footholds during primate locomotion in the wild and presumably reduce roll [47]. Although previous work has investigated the effects of forced ‘laddering’ across a series of poles perpendicular to the direction of travel [48], no study has attempted to recreate a branching pattern with a central pole available along the direction of travel.

Many of these studies, and others, have also examined the effect of substrate differences on limb posture and force production, and the ways in which the animal adjusted footfall pattern or limb angles to moving on a sloped substrate (for a review see [49]). While such factors are not currently considered in our model of walking, they are areas ripe for future expansion. We hope that they can be incorporated into a more detailed model in the future.

Primates show more protracted forelimb postures, more retracted hindlimb postures, and more pronounced joint yields on poles or arboreal supports than on the ground [19, 50–55]. This has a number of potential effects that can drive footfall pattern: increased stride length and yield may increase contact time overall [51, 52, 54] and can also lead to changes in limb overlap [5, 7, 8] or swing time [56]. This issue is highlighted in Franz et al. [57] in their comparison of limb phase values on multiple substrates for multiple gaits across a variety of speeds.

There has been some speculation on the relationship between forces and gait patterns in primates. Cartmill et al. [7] considered impact forces associated with pitch and their effects on gait choice. Schmitt et al. [16] specifically modelled the vertical forces of ambling gaits, confirming that different gaits (walk, trot, gallop) showed different force patterns [58]. But at present, there is little reason to suspect that force and footfall pattern within a gait (in our model walking) are linked. Schmitt [50, 51] reported lower peak forces values on poles compared to the ground in primates, a pattern confirmed in later studies of additional primate taxa [55, 57, 59] and arboreal marsupials [19, 60, 61]. Since arboreal marsupials showed DS gaits and relatively lower forelimb peak vertical forces, and more terrestrial ones used LS gaits and had relatively higher peak forelimb forces [60, 62], it seemed logical that footfall pattern and forces might be linked. In apparent support of that, Schmitt [59] found that one primate that consistently used LS gaits, the common marmoset, also had relatively high forelimb peak force. Wallace and Demes [11] also showed an association between decreased peak forelimb forces and DS gaits. Nonetheless, there is no mechanical argument in these studies as to why footfall sequence and force distribution should be linked. Moreover, studies of lorises [63, 64] have shown that force, limb position, and footfall timing can vary independently, effectively obviating this link.

Although studies of mediolateral forces are rare, those that have been carried out provide further emphasis concerning stability in our model. Schmitt [59] and Carlson et al. [65] both reported changes in magnitude and orientation of mediolateral forces in animals moving on raised pole substrates compared to the ground, confirming that roll and mediolateral stability are important factors in arboreal locomotion, a problem also considered in depth by Lammers [66].

Since much arboreal locomotion occurs on inclined substrates, movement on sloped substrates is another potentially very valuable area of study, particularly because the distribution of fore- and hindlimb force application is switched in primates compared with non-primate mammals (for example see [57]), and sloped substrates provide a natural experimental model. However, at present, the data provide a complex picture [34, 38, 40, 67–69].

It is also worth discussing the special problem of trotting gaits. While our model concentrates on explaining the selection of sequenced footfall patterns in mammals, the frequent use of the walking (with no aerial phase) or running trot by non-primate mammals and its almost complete absence in primates (see Schmitt et al. [16] for a review), makes it an interesting topic and worth mentioning here. Many studies have reported on walking trots (gaits with limb phase values of 50% +/− 5%) in primates and both walking and running trots in other animals on various substrates (see Cartmill et al. [5, 7]; Schmitt et al. [16] for a review). Some of those have found substrate-dependent differences and made links between trots and energetic costs. For example, Shapiro and Young [35] found that sugar gliders would trot consistently on poles. Low energetic recovery values were also noted during pole trotting in gray short-tailed opossums [70].

Several theories seek to explain the use of trotting gaits on arboreal substrates in non-primates. From their work on rats, Schmidt and Fischer [71] stressed the importance of coordinated lateral displacements of the thorax and pelvis in maintaining balance while moving on thin branches with a lateral-sequence footfall pattern. Lammers and Zurcher [72] found that the Siberian chipmunk–at least at faster speeds–relied upon dynamic stability to maintain balance on pole substrates. This strategy was also observed by Galvez-Lopez et al. [73] in dogs, but not in cats under the same experimental conditions, which is consistent with the idea that dogs and cats use different strategies and priorities in gait selection. This is an area ripe for future study, especially since the neurological basis of LS and DS footfall patterns remains unclear, along with the origins of DS gaits in primates. Work with a non-primate model moving on a side-branched pole such as the one used in the present study would likely be very enlightening.

### Hypotheses

**Fig. 3:**
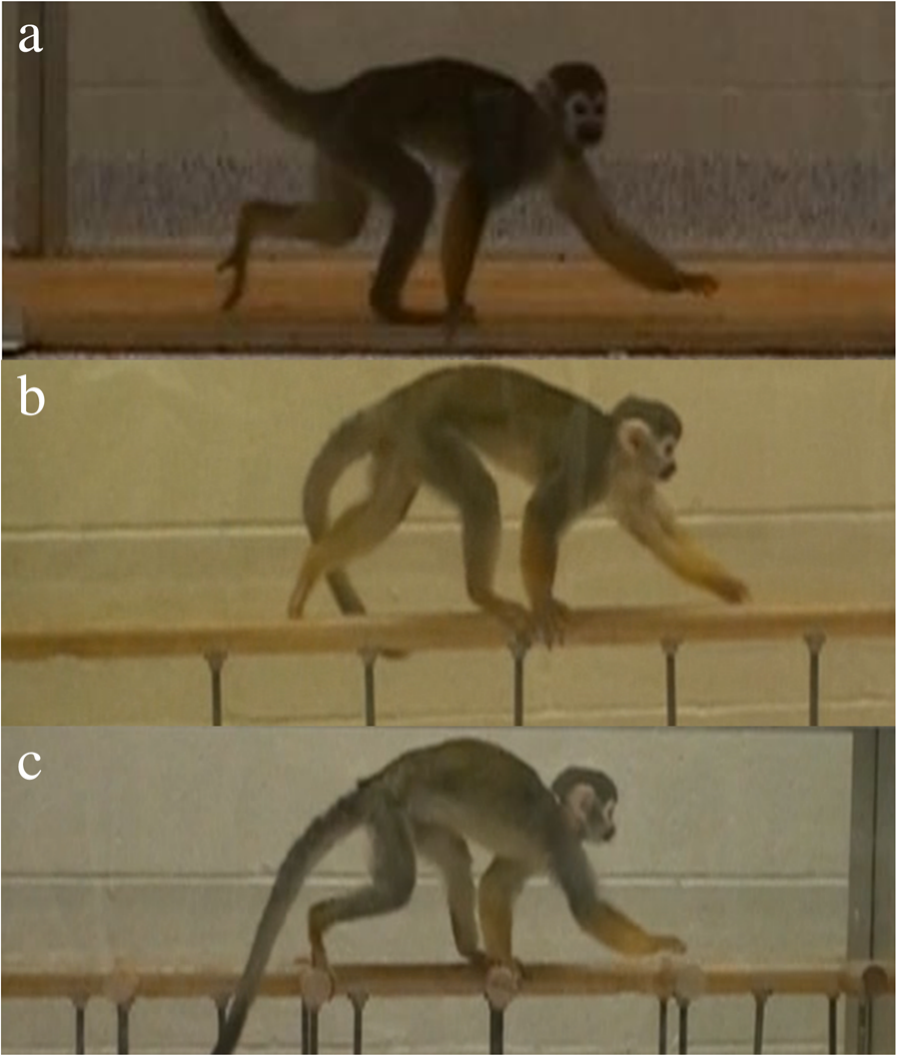
The three substrate types **a**) ground, **b**) straight pole, **c**) side-branched pole

Our model asks how much of the variance in footfall timing can be explained by cost (as predicted by Griffin et al. [14] and Usherwood et al., [17]) versus roll and pitch [7]. Under these conditions, we hypothesize that most of the variation can be explained by these factors without explicit consideration of additional variables, including limb compliance and peak loads. We test for effects of pitching and rolling moments in driving tendencies for animals to choose certain limb phase values predicted by the model by observing the frequency and footfall timings of LS and DS walks from two squirrel monkeys (*Saimiri sciureus*), a primate which reportedly uses variable footfall patterns [38]. While this sample is very small, it is presented as an initial test of the performance of the model, not as a definitive statement about gait choice in this or other primates. Animals were tested on three different substrates: (a) flat surface (equivalent to the ground), (b) raised, narrow horizontal pole, and (c) raised, side-branched horizontal pole (Fig. 3a-c), representing respectively:

1. A simple roll-*reducing* environment
2. A simple roll-*inducing* environment
3. A complex roll-*reducing* environment

We hypothesize that if LS gaits are truly disadvantageous due to associated rolling moments, they will be observed at a lower frequency on the roll-*inducing* narrow straight pole substrate than either the flat surface or side-branched pole.

Second, we hypothesize that, following the constraints outlined in the model (Fig. 2): limb phase values on the flat surface will tend toward those timings that are effective in minimizing energetic cost while avoiding limb interference [14, 17, 36], reaching values somewhere between 15 and 22% or 65 and 72%. In contrast, footfall timing on the pole substrates will be more centrally located between singlefoot and simultaneous gaits due to the added constraints of arboreal locomotion: minimizing fore-aft pitching moments, and rolling moments, particularly outside of the ‘zone of minimal bipedality’.

## Methods

To examine the predictions of tendencies in footfall selection by quadrupeds on terrestrial and arboreal supports from our model, data were collected from two adult male *Saimiri sciureus* (Table 1). All data collection methods were approved and monitored by the Duke University Institutional Animal Care and Use Committee.

**Table 1:** Subject details and distribution of walking steps on each of the three substrates

	Body mass (kg)	Ground	Straight pole	Sidebranched pole
B	0.86	42	35	84
L	0.82	7	45	2
Total steps		49	80	86

Animals were habituated to the experimental set-up with several training days. Data were collected after animals appeared at ease with the experimental conditions. Animals were encouraged to move freely along a given substrate and were rewarded with food treats when a bout along the entire length of the support was completed. Following habituation, animals were filmed in lateral view with a digital Sony Handycam (Sony USA, New York, NY) at 120 Hz while they walked at natural speeds on three wooden substrates coated with a layer of sand and varnish to increase friction. Three substrates were used: (a) 2.4 m × 0.6 m flat board; (b) 2.4 m straight pole, (3.1 cm in diameter) securely bolted to a flat base; and (c) 2.4 m pole (3.1 cm diameter) with 3.1 cm side branches of the same diameter running perpendicular to the direction of travel every 21 cm along its length.

Following the methods of Cartmill et al. [5], the timing of touchdown events for each limb were recorded for an entire stride using DLT dataviewer (DLTdv3 [74]) to allow direct comparison with earlier work. The resulting time values were exported to custom scripts in MATLAB (The Mathworks, Natick, MA) for which values of limb phase and duty factor were calculated. In addition, videos were calibrated for pixel:metric length values in x and y directions using calibration items recorded during data collection sessions. Statistics were also performed in MATLAB. DS limb phase values were found to be non-normally distributed via Kolmorogov-Smirnov testing, and regression analysis did not show a consistently significant relationship between speed and duty factor, limb phase and speed, or limb phase and duty factor across substrates (see results section below). Therefore, groups were compared by Kruskal-Wallis non-parametric ANOVAs and pairwise Wilcoxon sum rank tests. A chi-square test was also performed to examine the distribution of footfall sequences between substrates, with the expected values being calculated from the total number of observed LS footfall patterns, and distributed with equal frequency across all three substrates.

## Results and application to model

The two animal subjects (subject B and subject L) did not walk the same number of times on all substrates. Walking steps were most evenly distributed between the two animals on the straight pole (Table 1). Although subject L was generally less inclined to walk on the flat substrate and side-branched pole, values obtained for these steps fall within the range of those of subject B.

Lateral-sequence gaits were observed in one subject only (Subject B) and only on the flat board and side-branch pole, both of which are roll-reducing substrates. The observed distribution of LS strides differed significantly from the distribution expected to occur by chance (Table 2; χ^2^ = 16.3; *p* < 0.05). Within the two roll-reducing substrates (flat surface and straight pole), the data are skewed towards a greater proportion of LS gaits on the flat surface than on the side-branched pole. Therefore, LS gaits appear to be more common on the flat surface than on other substrates.

**Table 2:** Distribution and of lateral-sequence (LS) walking steps observed in subject B (no LS walks were observed for subject L), and their expected distribution (to the nearest whole number) in the absence of bias. The distribution of LS walks is significantly different from that expected in the absence of bias

B	Total steps	Percentage (%)	LS observed	LS expected
Ground	42	16.7	7	2
Straight pole	35	0	0	2
Sidebranched pole	84	2.4	2	5

Chi square value = 16.3, significance threshold (5%) = 5.991

Mean duty factors (± one standard deviation) were 58 ± 4.6% on the ground, 58.7 ± 3.2% on the pole, and 60.7 ± 2.4% on the side-branched substrate. Duty factor was significantly greater on the side-branched pole than it was on the ground and straight pole (*p* < 0.0001). Speed values were 1.06 ± 0.23 ms^− 1^ on the ground, 0.99 ± 0.13 ms^− 1^ on the straight pole and 1.04 ± 0.13 ms^− 1^ on the side-branched pole, with only the straight and side-branched pole substrates differing significantly in speed values.

While correlations between speed and duty factor were significant for all substrates and across the combined dataset (*p* < 0.01 in all cases), the amount of variation explained varied by substrate. On the ground, 62% percent of the variation in the speed versus duty factor relationship was explained by their co-dependence. For the straight pole and side-branched pole, only 22 and 26% of the variance in duty factor was explained by speed respectively. It is worth noting that an increase in limb compliance in animals moving on pole substrates is known to influence contact time [51] and may explain the low correlations between duty factor and speed. This complex relationship may explain why the overall pattern of correlations between both limb phase and duty factor, and speed and duty factor was difficult to ascertain. Significant correlations were found only between limb phase and duty factor on the side-branched pole (*p* = 0.002, r^2^ = 0.11) and between limb phase and speed on the straight pole (*p* = 0.00003, r^2^ = 0.20). Because not all associations were significant and the slopes of these relationships varied, an ANCOVA was inappropriate; therefore, groups were compared by a Kruskal-Wallis test as a non-parametric alternative to an ANOVA.

**Fig. 4:**
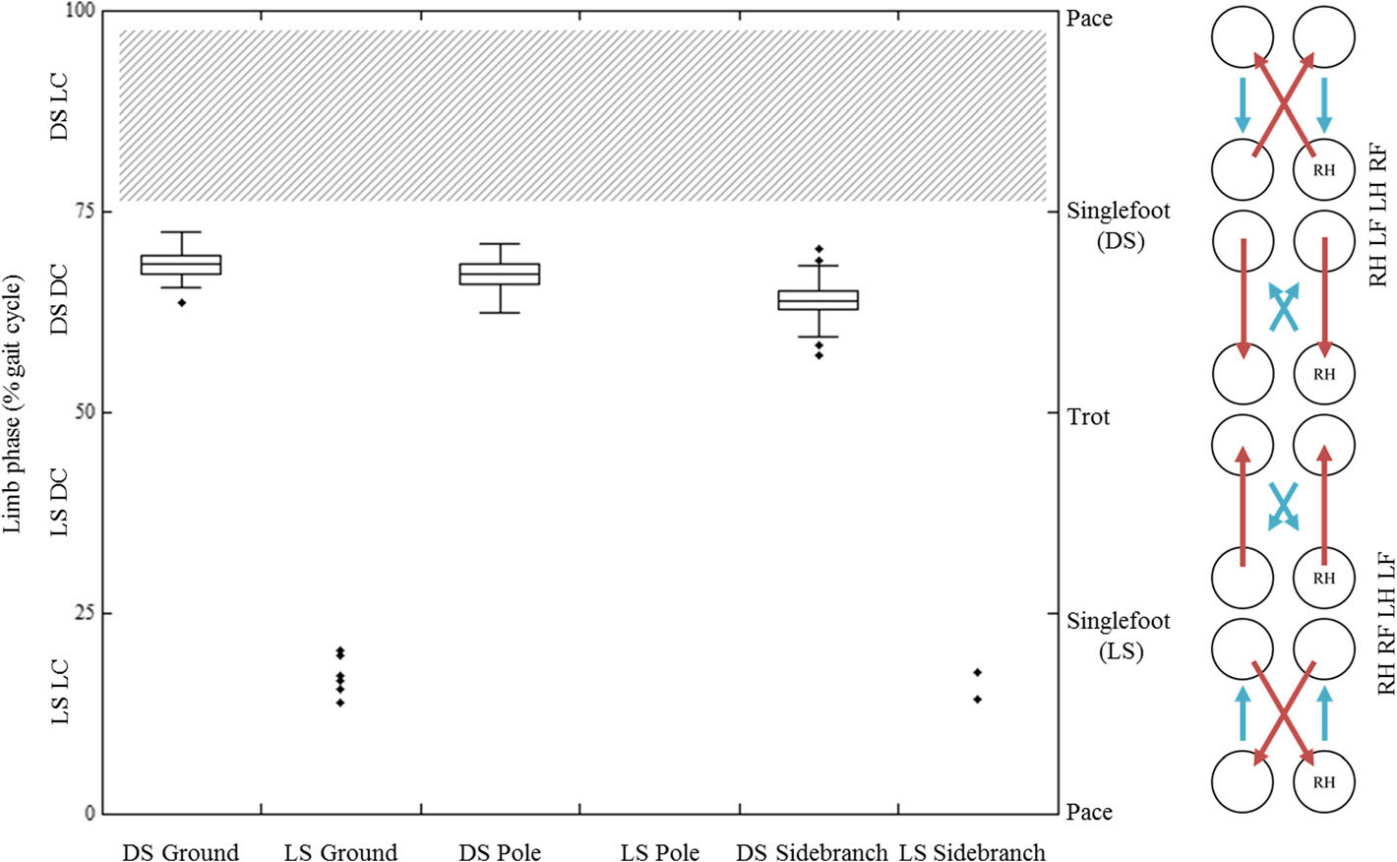
Limb phase data from walking steps on each substrate type in our sample of *Saimiri sciureus*. Box plots indicate maximum, minimum, median and quartile values for each substrate. LS: lateral sequence, DS: diagonal sequence, LC: lateral couplet, DC: diagonal couplet. No gaits were observed within the limb phase range of LSDC gaits in these animals, no animal has yet been recorded using a DSLC gait. On the footfall diagrams arrows indicate the direction of weight transfer. The direction of the arrow moving from the right hind foot ‘RH’ indicates sequence (LS or DS), direction of the short arrow indicates couplet type (LC or DC). Arrow length indicates whether the delay between touchdowns is short (less than 25% of a stride) or long (greater than 25% of a stride)

Within DS gaits limb phase values are relatively high, but are within the range of DS values observed for other primates and arboreal marsupials (see Discussion section below). Limb phase differs significantly between substrates (*p* < 0.001, Fig. 4), with mean values (± one standard deviation) for DS gaits of 68.25 ± 1.66% on the ground, 67.08 ± 1.87% on the straight pole, and 63.85 ± 2.32% on the side-branched pole. Although the differences are small in these preliminary data, pairwise testing shows that all three groups of limb phase values are significantly different, with *p* values below 0.001 for each pair.

## Discussion

One central prediction of our model is that DS gaits will be more common on narrow arboreal supports because of their roll-resistant properties. Of the 215 walking trials analysed, only nine LS gaits were observed. They were observed only in one of the two subjects, and only on the flat surface and side-branched pole, substrates that reduce the potential for body roll. The remainder of the steps were DS gaits with limb phase values over 50% similar to those observed in other primates such as *Sapajus* [11], *Ateles*, *Daubentonia,* and *Eulemur* [5, 7, 8], other samples of squirrel monkeys [39, 75], and arboreal mammals, including woolly opossums and kinkajous [23, 24].

As mentioned at the outset, it is not our goal to review arguments about the presence of DS gaits in primate versus non-primate mammals and its implications for primate evolution. This is an interesting and important area that is already well covered (for the highlights of this debate see [3, 5–7, 10, 32–36]). However, our data do suggest that LS gaits are more likely to be observed on roll-reducing substrates. Still, we also recognize that non-primate mammals moving on small poles [35] do use limb phase values below 50% (LS gaits). In addition, young primates and those on flexible supports will also sometimes use limb phase below 50% [9, 36, 41, 49] and, as Vilensky and Larson [32] noted, several species, including the squirrel monkey, appear to be able to use limb phase values above and below 50% with equal facility. Nonetheless, the overall pattern from our data and those of others in conjunction with the model developed here lends further support to the argument that adopting a DS or LS gait is related at least in part to problems of roll [5, 7, 8]. Within the few lateral sequence trials obtained, results are within the range of values found for other primates [39], arboreal marsupials [23], the echidna [76], and dogs and horses using non-pacing LSLC gaits [14, 17, 20]. This is consistent with these animals minimizing the energetic costs of locomotion via pendular exchange mechanisms when mediolateral stability demands are low.

To test model performance, we used two substrates that allowed the animals to place their feet in a wider gauge than that possible on the straight pole, potentially reducing rolling moments about the substrate. One of those substrates–the flat board–is simple and allows free foot and hand placement in all directions, while the other–the side-branched pole–is complex, and allows free placement on or to either side of the central pole, but dictates specific anteroposterior locations for foot and hand placement in steps of wider gauge. Of the two roll-reducing substrates, limb phase values are significantly greater on the flat surface than on the side-branched pole, though the absolute differences are small. Values on the roll-inducing straight pole are much closer to those on the flat surface. This result, though preliminary and representing small differences, may suggest that, within DS gaits, limb phase values, while related to some degree to the challenges imposed by rolling moments, may also be associated with broader issues of substrate complexity such as pitching moments, as outlined in Fig. 2c. As the flat surface is the least restrictive substrate in terms of footfall positioning, we would expect to see gait sequences less geared towards minimizing pitch and roll. Steps on the flat surface tend towards maximizing energy exchange, approaching singlefoot values as predicted by Griffin et al. [14], Usherwood et al. [17], and detailed in our model (Fig. 2). While this dataset is preliminary, and the absolute differences between values are very small, the model seems to hold up well to initial testing. This opens up the possibility for the addition of future data to better test the biological significance of these parameters, and possible expansion of the model to further contributing factors.

The data from the side-branched poles demonstrate broader aspects of flexibility in primate gait choice. Steps on the side-branched pole encompass the greatest range of limb phase values (Fig. 4). This is likely due to the animal having a wider range of possible ‘substrates’ available for individual footfalls, with each foot landing on one of three separate substrate types: straight pole, side branch, or junction point between side branch and straight pole. Side branches may present extra challenges related to forward pitch. On this substrate, animals may face increased pitching moments. A shift toward more simultaneous gaits with contralateral bipods may help reduce the risk associated with those pitching moments. In our study, the even spacing of side branches allowed for a complex but predictable substrate. Future studies should consider different side branch configurations. This would help to clarify the circumstances in which animals may choose to step on or skip over any given branch, increasing flexibility in stride length. Natural environments provide much greater substrate complexity than the straight and side-branched poles studied here, and a wider range of values should be expected from free-ranging animals. Indeed, comparable data collected on squirrel monkeys in naturalistic conditions do show a broader range of limb phase values within the region of the DSDC footfall pattern [39].

## Conclusions

We provide a model that examines two key factors thought to influence footfall timings in quadrupedal mammals, specifically how footfall timing will be impacted by constraints of energy expenditure and balance on different substrate types. This model predicts that limb phase values which minimize energy costs will be most common on the flat, ground-like surface, and those that moderate pitch and roll will be more common on arboreal supports. The performance of the model is examined by comparing predicted tendencies to footfall timings in a small dataset of squirrel monkey walking strides, a primate species that is known to use a wide range of limb phase values. These strides covered both lateral-sequence and diagonal-sequence footfall patterns, and were observed on flat surfaces and raised poles with or without side branches. The expectations of the model were supported by these data, showing that on narrow substrates animals will choose gaits that reduce roll, even though they may increase locomotor costs. On flat or other roll-reducing surfaces, however, animals use limb phases that maximize energetic efficiency. The model appears to perform well and hopefully lays the groundwork for further data collection in other species, and for the addition of other factors into the model. With that continued validation and refinement process, we hope that this model will further our understanding of footfall patterns and gait selection in mammals.

## Abbreviations

COM: Center of mass
DC: Diagonal couplet
DS: Diagonal sequence
KE: Kinetic energy
LC: Lateral couplet
LF: Left fore foot
LH: Left hind foot
LS: Lateral sequence
PE: Potential energy
RF: Right fore foot
RH: Right hind foot
